# Rate of presence of 11 thoracic vertebrae and 6 lumbar vertebrae in asymptomatic Chinese adult volunteers

**DOI:** 10.1186/s13018-018-0835-9

**Published:** 2018-05-23

**Authors:** Ying-zhao Yan, Qing-ping Li, Cong-cong Wu, Xiang-xiang Pan, Zhen-xuan Shao, Shao-qing Chen, Ke Wang, Xi-bang Chen, Xiang-yang Wang

**Affiliations:** 1 Department of Orthopaedic Surgery, Zhejiang Spine Surgery Centre, The Second Affiliated Hospital and Yuying Children’s Hospital of Wenzhou Medical University, 109 Xueyuanxi Rd, Wenzhou, 325027 Zhejiang China; 2 Department of Radiology, The Second Affiliated Hospital and Yuying Children’s Hospital of Wenzhou Medical University, 109 Xueyuanxi Rd, Wenzhou, 325027 Zhejiang China

**Keywords:** 11 thoracic vertebrae, 6 lumbar vertebrae, Wrong-site surgery, Spinal alignment, Asymptomatic volunteers

## Abstract

**Background:**

An increasing number of studies on spinal morphology in asymptomatic Asian and Western patients have been reported. Variation in spinal anatomy among patients is considered as the cause of wrong-level surgery in up to 40% of cases. The present study examined the rate of presence of 11 thoracic vertebrae and 6 lumbar vertebrae in 293 asymptomatic Chinese adult volunteers.

**Methods:**

From May 27, 2016, to November 11, 2017, a cohort of 325 asymptomatic Chinese adults meeting the study exclusion criteria was recruited. The radiographs were examined by a spine surgeon and a radiologist to assess the number of thoracic and lumbar vertebrae.

**Results:**

In total, 293 volunteers were included in this study: 17 (5.8%) had 11 thoracic vertebrae, and 16 (5.5%) had 6 lumbar vertebrae. Among all volunteers, 12 (4.1%) had 7 cervical vertebrae (C), 11 thoracic vertebrae (T), and 5 lumbar vertebrae (L); 5 (1.7%) had 7C, 11T, and 6L; and 11 (3.8%) had 7C, 12T, and 6L. There was no difference between the findings of the spine surgeon and the radiologist.

**Conclusions:**

For the first time, this study describes the rate of presence of 11 thoracic vertebrae and 6 lumbar vertebrae in 293 asymptomatic Chinese adult volunteers. Variations in the number of thoracic and lumbar vertebrae tend to be ignored by spine surgeons. We encourage spinal surgeons and researchers to be aware of such variations when performing thoracic- and lumbar-level surgery and assessing spinal alignment and parameters.

## Background

Studies on the spinal morphology and alignment of asymptomatic Asians and Westerners are being reported with increasing frequency [1–9]. However, many such studies do not consider variations in the number of vertebrae, which can also lead to wrong-site surgery. It has been reported that as many as 50% of spinal surgeons have performed incorrect vertebral level surgery during their careers [10–12]. Variation in patient anatomy is considered the cause in up to 40% of cases of wrong-level surgery [11, 13].

Many surgeries are carried out in the spinal center of our hospital each year, primarily for fracture reduction, discectomy, and scoliosis correction. To avoid wrong-level surgery, we preoperatively check whole-spine images with cephalocaudal enumeration. The aims of this study were to describe the rate of presence of 11 thoracic vertebrae and 6 lumbar vertebrae in Chinese asymptomatic adult volunteers and to encourage spinal surgeons to be aware of the variations in the numbers of thoracic and lumbar vertebrae when performing thoracic- and lumbar-level localization and measuring spinal parameters.

## Methods

### Subject enrollment and data collection

This study received institutional review board approval and followed the principles of the Declaration of Helsinki. From May 27, 2016, to November 11, 2017, a cohort of 325 asymptomatic Chinese adults was recruited who were aged above 18 years and satisfied the following exclusion criteria: (1) lameness or unequal length of the lower limbs; (2) apparent scoliosis; (3) history of trauma of the spine, pelvis, or lower extremity; (4) history of hip or knee arthroplasty and spine, pelvis, or lower-limb surgery; (5) complaints of back pain, neck pain, or limb numbness caused by degenerative diseases of the spine, such as disc herniation, spinal canal stenosis, and lumbar spondylolisthesis; (6) strabismus or torticollis affecting balance; (7) history of neuromuscular disorders or congenital abnormalities; or (8) pregnancy or preparation for pregnancy.

Informed consent was obtained from each volunteer prior to the enrollment in this trial.

The volunteers were entitled to a free full-spine photograph and X-ray report, including of the chest, lungs, spine, and abdomen, in return for their participation.

### Radiographic analysis

Full-spine standing anteroposterior and lateral radiographs were acquired for all volunteers with their arms in the fists-on-clavicles position. The radiographs were examined by a spine surgeon and a radiologist who had independently reviewed several hundred whole-spine images prior to this review.

In the posteroanterior view, the top rib was regarded as the first thoracic level, and enumeration proceeded caudally. Thoracic vertebrae were identified according to the corresponding rib attachments. All vertebrae with rib attachments, including the bilateral or unilateral ribs, were counted as thoracic vertebrae. A vertebra was considered to be at the lumbar vertebrae level only if it was not attached to the ribs.

Continuing caudally, the first lumbar vertebra was that following the last thoracic vertebra. When there was a complete vertebra between L5 and the sacrum, and well-formed disc material extending between the vertebra and sacrum, the vertebral body was defined as L6. Lumbosacral transitional vertebrae (LSTV) were defined based on previous literature [13, 14], i.e., one or both transverse processes attached to the sacrum through incomplete or complete osseous fusion or via a diarthrodial joint.

### Statistical analyses

The SPSS statistical software package (ver. 19.0; SPSS Inc., Chicago, IL, USA) was used for the statistical analyses. Demographic data conforming to a normal distribution were expressed as means ± standard deviation. Patient demographic characteristics including age, weight, height, and body mass index (BMI) were compared using independent samples *t* tests. Variables expressed as frequencies were compared using the chi-squared test. *p* values less than 0.05 were deemed to indicate statistical significance.

## Results

**Fig. 1:**
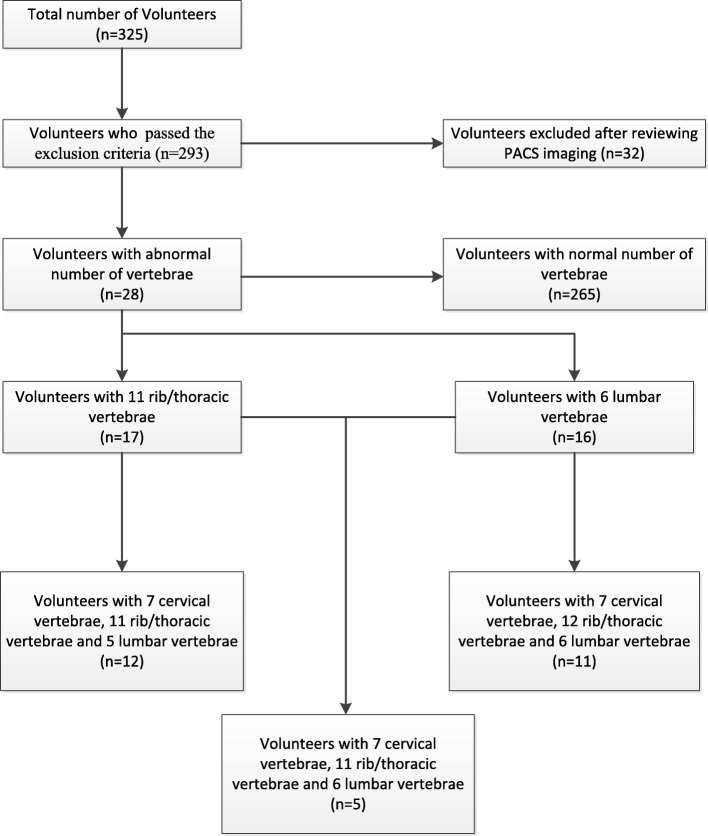
A schematic illustrating the research process and distribution of asymptomatic volunteers with atypical numbers of vertebrae

Thirty-two volunteers had missing X-ray images or did not meet the exclusion criteria (Fig. 1). In total, 293 volunteers were included in this study.

The spine surgeon confirmed that two volunteers had only four lumbar vertebrae, whereas the radiologist believed there were no four-lumbar vertebrae volunteers. In addition, differences between the surgeon and radiologist were seen in the results of the vertebral body count, even though they both used the same method (see Table 1).

**Table 1:** The results of a variable number of vertebrae by spine surgeon and radiologist

	Spine surgeon	Radiologist	After joint reconfirmation
7C + 11T + 5L	11 (3.8%)	12 (4.1%)	12 (4.1%)
7C + 11T + 6L	12 (4.1%)	11 (3.8%)	5 (1.7%)
7C + 12T + 4L	2 (0.7%)	0	0
7C + 12T + 6L	12 (4.1%)	10 (3.4%)	11 (3.8%)
7C + 12T + 5L	256 (87.4%)	260 (88.7%)	265 (90.4%)
Total	293	293	293

There was no statistical difference between spine surgeon and radiologist

*C* cervical vertebrae, *T* thoracic vertebrae, *L* lumbar vertebrae

Differences were reviewed by the surgeon and radiologist, and a consensus was obtained in all cases. Twenty-eight (9.6%) of the volunteers had an atypical number of thoracic and/or lumbar vertebrae. Seventeen (5.8%) volunteers had 11 thoracic vertebrae, and 16 (5.5%) had 6 lumbar vertebrae. An LSTV was present in nearly all patients who had an atypical number (i.e., six) of lumbar vertebrae (15 of 16; 93.8%). In total, 5 (1.7%) of the 293 volunteers had an atypical number of both thoracic (11) and lumbar (6) vertebrae. No volunteer had 13 thoracic vertebrae and/or 4 lumbar vertebrae (Fig. 1).

**Fig. 2:**
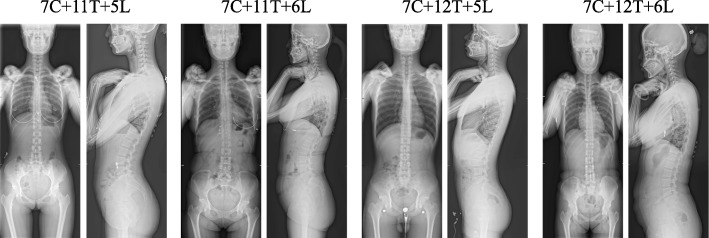
Full-spine X-ray images of each group

Based on the above results, the volunteers were divided into four groups: group 7C + 11T + 5L, group 7C + 11T + 6L, group 7C + 12T + 5L, and group 7C + 12T + 6L. The X-ray images of each group are shown in Fig. 2.

The patient demographic data are included in Table 2. The volunteers had a mean age of 40.8 ± 12.8 years (range 24 to 74 years), with a mean height of 164.0 ± 7.0 cm (range 148 to 186 cm), weight of 61.1 ± 9.6 kg (range 40 to 95 kg), and BMI of 22.6 ± 2.8 kg/m^2^ (range 16.2 to 32.9 kg/m^2^). In total, 111 (38%) volunteers were male, and 182 (62%) were female. The differences in weight, height, and BMI between group 7C + 11T + 5L, and group 7C + 12T + 5L were statistically significant (*p* < 0.05).

**Table 2:** Demographic characteristics of different groups

	Group				Total
	7C + 11T + 5L	7C + 11T + 6L	7C + 12T + 6L	7C + 12T + 5L	
Number	12	5	11	265	293
Male/female	1/11	1/4	8/3	101/164	111/182
Age	35.9 ± 14.2 (23~70)	42.8 ± 9.9 (29~53)	40.2 ± 13.9 (24~63)	40.9 ± 12.8 (20~74)	40.8 ± 12.8 (20~74)
Weight (kg)	51.6 ± 8.7** (41~67)	58.7 ± 2.7 (55~62)	60.5 ± 8.9 (49~75)	61.5 ± 9.6 (40~95)	61.1 ± 9.6 (40~95)
Height (cm)	160 ± 6.0* (155~175)	163 ± 3.8 (158~167)	167 ± 8.0 (154~180)	164 ± 7.0 (148~186)	164 ± 7.0 (148~186)
BMI (kg/m^2^)	19.9 ± 2.2** (17.1~22.6)	22.1 ± 1.5 (21.2~24.8)	21.6 ± 2.6 (16.9~25.3)	22.8 ± 2.7 (16.2~32.9)	22.6 ± 2.8 (16.2~32.9)

The range is shown in parentheses; “7C + 12T + 5L” stands for volunteers with normal number of vertebrae

*C* cervical vertebrae, *T* thoracic vertebrae, *L* lumbar vertebrae

*Compared with group 7C + 12T + 5L, *p < 0.05*

**Compared with group 7C + 12T + 5L, *p* < 0.01

## Discussion

A previous study reported that approximately 10–17.4% of adults have some form of spinal abnormality, the most common of which is the presence of L6 [1, 5]. Another large-scale study reported that people with LSTV account for 4–30% of the general population [15]. In addition, approximately 5–8% of “normal” individuals lack a pair of ribs/thoracic vertebrae [16], while additional ribs are sometimes considered as normal variants. However, we found no study of normal individuals having 11 thoracic vertebrae combined with 6 lumbar vertebrae. This study was a cross-sectional analysis of the rate of presence of 11 thoracic vertebrae and 6 lumbar vertebrae among 293 healthy subjects, as visualized on full-spine standing radiographs. We found that 9.6% of the asymptomatic population had an atypical number of thoracic and/or lumbar vertebrae. Among all volunteers, 4.1% were included in the 7C + 11T + 5L group, 1.7% in the 7C + 11T + 6L group, and 3.8% in the 7C + 12T + 6L group. Thus, 5.8% of the volunteers had 11 thoracic vertebrae, and 5.5% had 6 lumbar vertebrae. We have to thank the reviewers of this article for helping us reconfirm the number of cases in group 7C + 11T + 6L. Our findings are consistent with those of the previous studies [15, 16]. However, the height and weight of the volunteers with 11 thoracic vertebrae and 5 lumbar vertebrae were significantly lower than those of the volunteers with a normal number of vertebrae. We believe that a reduction in the number of thoracic vertebrae has a great effect on body length and body size.

Studies on the spinal morphology and alignment are being reported with increasing frequency [1–9]. More than 80 articles published in the past 5 years were retrieved from the PubMed database by a search including the keywords “asymptomatic,” “spine,” and “alignment.” These studies measured spinal parameters, including occipitocervical alignment [2], cervicothoracic alignment [2, 7], spinopelvic alignment [5, 8], cervical parameters [6], thoracic parameters [6, 9], and lumbar parameters [4], and described their role in spinal balance or the diagnosis and treatment of spinal diseases. However, although these studies used various measures to reduce errors when measuring spinal parameters, such as the establishment of exclusion criteria, and performance of multiple measurements by various experts, many of them ignored the important question of whether spinal parameters are measured accurately when there is variation in the number of vertebrae among patients [1–9]. For example, Mizutani et al. did not explain in their Methods section how they accounted for an absence of thoracic vertebrae, and therefore, we are unsure of how they dealt with that situation [9]. Yokoyama et al. [5] described the rate of presence of six lumbar vertebrae among a Japanese population and regarded the sacrum below the LSTV as a marker to evaluate the spinopelvic alignment of six lumbar vertebrae. Although an intervertebral disc exists between the L6 vertebra and the inferior sacral vertebra, mobility between the L6 vertebra and sacrum may be restricted [17]. When measuring the thoracic parameters of 11 thoracic vertebrae individuals, replacing T12 with T11 is the first intuition. However, if both 11 thoracic vertebrae and L6 are present, and L6 did not originate in S1, the superfluous first lumbar vertebra may be the last thoracic vertebra lacking ribs. In such cases, it seems appropriate to replace T12 with L1. Therefore, we suggest that, when collecting spinal alignment data from asymptomatic volunteers, exclusion criteria must be applied to exclude cases with an atypical number of vertebrae, even though these can account for 10–30% of all patients [5]. A spinal alignment database specifically pertaining to cases with an atypical number of vertebrae should be established. We remain skeptical of the article comparing global spinal alignment and balance between patients with atypical and normal numbers of vertebrae [5].

Wrong-level surgery is a sensitive and serious event for both the patients and the spine surgeons. Although over 50% of surgeons have performed wrong-level surgery during their career, many spinal surgeons still believe that it is completely avoidable [12]. Certain factors, including atypical anatomy, have been considered responsible for wrong-site spine surgery. Based on an analysis of 65 spinal surgery lawsuits, Goodkin et al. demonstrated that mistakes may arise due to omission or misunderstanding of imaging studies performed before or during surgery [18].

An atypical number of vertebrae and the presence of LSTV may hamper accurate assessment of spinal anatomy. Approximately 5.5% of our asymptomatic volunteers showed an atypical L6 variation, in 93.8% of cases caused by LSTV. During the last 10 years, only one wrong-level surgery was conducted at our spinal surgery center among more than 5000 surgeries. In that patient, who was scheduled for L5/S1 segment surgery, we misperformed a decompression between L6 and the sacrum due to the presence of L6. The patient was not satisfied with the level of pain relief achieved after surgery; thus, as a remedial measure, selective nerve root block of L5/S1 was performed after communicating with the patient. Previous studies have also noted the role of LSTV in wrong-level discectomies. In some series, wrong-level discectomies due to variations in the number of vertebrae accounted for 40–71% of all procedures [10, 11]. If 11 thoracic vertebrae are present, the ability to determine the surgical level before or during surgery based on cephalocaudal enumeration will be affected by changes in the thoracic vertebrae. Although researchers have proposed several lumbar localization methods for thoracolumbar surgery [12, 19], Longo suggests that further strategies are needed to reduce the risk of wrong-level surgery [20]. Mody et al. [10] made three recommendations to surgeons: direct communication with the patients before surgery, marking of predetermined sites, and use of verification radiographs.

In this study, there was a difference between the spine surgeon and radiologist in the ability to discriminate among vertebral variations, although the difference was not statistically significant. Considering the high (9.6%) incidence of an atypical number of vertebrae, we recommend that spinal surgeons should not rely on the radiologist’s report alone; ideally, the radiologist and surgeon should preoperatively determine the number of vertebrae together. More conveniently, the surgeon can associate the preoperative findings with the intraoperative X-ray films.

To our knowledge, this is the first study of asymptomatic patients showing variation in the number of thoracic and lumbar vertebrae. However, some weaknesses of the study should be acknowledged. First, the sacrum is tilted at about 40° in full-spine upright radiograph, so that it is difficult to evaluate L6 or LSTV. Regardless of how carefully we examined these radiographs, there could be a certain amount of misdiagnosis. Besides, we could not confirm that the vertebra variants were indeed LSTV by computed tomography (CT). So, we have to make a serious statement about the potential misdiagnosis of T12, L6, or LSTV in upright radiographs. It must be clear to all readers of this article that the data provided above is just a bit closer to the truth. Second, we cannot be sure that the incidence of variations in the number of thoracic or lumbar vertebrae in our limited sample is representative of the rate of such variations among the general population of eastern China; this remains to be confirmed by other researchers. Third, we did not include any patients with cervical ribs, 4 lumbar vertebrae, or 13 thoracic vertebrae. As expected, our selection criteria excluded these particular vertebrae variants. The rate of presence of cervical ribs varies from 0.05 to 8% in the general population, and they are rarely symptomatic in early childhood; however, in older children and adults, thoracic outlet syndrome or aneurysm formation can occur [16, 21, 22]. Supernumerary ribs, seen in trisomy 21 syndrome, are rarely seen as a normal variant [16]. Thus, in choosing adult asymptomatic volunteers, we excluded such variations.

## Conclusion

For the first time, this study reported the rate of presence of 11 thoracic vertebrae and 6 lumbar vertebrae in 293 asymptomatic Chinese adult volunteers. We found that 4.1% of the patients were included in the 7C + 11T + 5L group, 1.7% in the 7C + 11T + 6L group, and 3.8% in the 7C + 12T + 6L group. These variations tend to be ignored by spine surgeons; thus, we suggest that spinal surgeons and researchers should be aware of the variations in the number of thoracic and lumbar vertebrae when performing thoracic- and lumbar-level surgery and when assessing spinal alignment and parameters.

## Abbreviations

C: Cervical vertebrae
L: Lumbar vertebrae
LSTV: Lumbosacral transitional vertebrae
T: Thoracic vertebrae
